# Obsessive-compulsive disorders as a result of cervical spine injuries in geriatric patients

**DOI:** 10.1192/j.eurpsy.2026.11598

**Published:** 2026-07-31

**Authors:** N. Syrmos

**Affiliations:** Aristotle University, Thessaloniki, Greece

## Abstract

**Introduction:**

Introduction- Cervical spine neuropsychiatric results are important causes of morbidity. Among of theme are the obsessive-compulsive disorders (OCD).

**Objectives:**

Aim-Aim of this study is to present cases with obsessive-compulsive disorders as a result of cervical spine injuries in geriatric patients

**Methods:**

Material-Methods-10 patients are presented.9 males (90%) and 1 females (10%) Range of age between 70-80years old . All of theme (10-100%) were evaluated, with clinical and radiological exam(ct,mri) plus diagnostic tests for Obsessive-compulsive disorders

**Results:**

Results-Good results in 6 cases (60%) and moderate in 4 (40%). Collaboration between various medical disciplines is essential in order to achive optimal results and clarify the theory,the etiology, and treatment effectiveness..

**Conclusions:**

Conlusions-Premorbid status can be obtained by structured psychiatric interviews, and traumatic brain lesions can be defined with advanced neuroimaging techniques.

**Disclosure of Interest:**

None Declared

